# Evaluation of a High-Intensity Green Fluorescent Protein Fluorophage Method for Drug- Resistance Diagnosis in Tuberculosis for Isoniazid, Rifampin, and Streptomycin

**DOI:** 10.3389/fmicb.2016.00922

**Published:** 2016-06-17

**Authors:** Xia Yu, Yunting Gu, Guanglu Jiang, Yifeng Ma, Liping Zhao, Zhaogang Sun, Paras Jain, Max O'Donnell, Michelle Larsen, William R. Jacobs, Hairong Huang

**Affiliations:** ^1^National Clinical Laboratory on Tuberculosis, Beijing Key Laboratory for Drug-Resistant Tuberculosis Research, Beijing Chest Hospital, Beijing Tuberculosis and Thoracic Tumor Institute, Capital Medical UniversityBeijing, China; ^2^Department of Microbiology and Immunology, Albert Einstein College of MedicineBronx, NY, USA; ^3^Division of Pulmonary, Allergy, and Critical Care Medicine, Columbia University Medical CenterNew York, NY, USA; ^4^Department of Epidemiology, Mailman School of Public Health, Columbia University Medical CenterNew York, NY, USA; ^5^Howard Hughes Medical InstituteChevy Chase, MD, USA

**Keywords:** tuberculosis, mycobacteriophage, drug resistance, diagnosis, *Φ*^2^*GFP10*

## Abstract

A novel method for detecting drug resistance in *Mycobacterium tuberculosis* using mycobacteriophage *Φ*^2^*GFP10* was evaluated with clinical isolates. The phage facilitates microscopic fluorescence detection due to the high expression of green fluorescence protein which also simplifies the operative protocol as well. A total of 128 clinical isolates were tested by the phage assay for isoniazid (INH), rifampin (RIF), and streptomycin (STR) resistance while conventional drug susceptibility test, by MGIT960, was used as reference. The sensitivities of *Φ*^2^*GFP10* assay for INH, RIF, and STR resistance detection were 100, 98.2, and 89.3%, respectively while their specificities were 85.1, 98.6, and 95.8%, respectively. The agreement between phage and conventional assay for detecting INH, RIF, and STR resistance was 92.2, 98.4, and 93.0%, respectively. The *Φ*^2^*GFP10*-phage results could be available in 2 days for RIF and STR, while it takes 3 days for INH, with an estimated cost of less than $2 to test all the three antibiotics. The *Φ*^2^*GFP10*-phage method has the potential to be a valuable, rapid and economical screening method for detecting drug-resistant tuberculosis.

## Introduction

Reduction in transmission of drug resistant tuberculosis (TB) and improved patient management requires timely diagnosis of drug resistant bacilli. Presence of drug-resistant *M. tuberculosis* bacilli is confirmed by genotypic and phenotypic drug susceptibility testing (DST). Genotypic approaches with short turnaround time are based on identification of well-known antibiotic resistance-conferring gene mutations. Despite active research, not all TB drugs can have genotypic drug-susceptibility testing since the genetic basis of resistance may be complex or incompletely characterized (Kruuner et al., 2003; Jain et al., 2011; Cui et al., 2013; Zhang et al., 2015). Phenotypic assays observe the bacterial response to antibiotics *in vitro* without limitation to any particular antibiotic, allele, or working mechanism although the phenotypic DST is often time consuming.The conventional DST, based on solid medium, takes about 4–6 weeks after the isolation of *M. tuberculosis*, on the other hand BACTEC MGIT960 requires 10–14 days after acquiring a positive culture. Therefore, it is imperative to outline a further rapid, accurate, inexpensive DST method for the diagnosis of drug resistance *M. tuberculosis*.

Phage based methods have been used for drug resistance detection of *M. tuberculosis* since about two decades ago, such as bacteriophage D29 (Wilson et al., 1997) and luciferase reporter phages (LRPs) (Jacobs et al., 1993). A novel, high-intensity mycobacteria-specific fluorophage (*Φ*^2^*GFP10*) was described recently with good results in pre-clinical evaluation of drug resistant tuberculosis (Jain et al., 2012). *Φ*^2^*GFP10* is an improved second generation fluorescent reporter phage which expresses *gfp* (green fluorescence protein) under the control of P_L_ promoter of mycobacterium phage L5 (Guo and Ao, 2012; Jain et al., 2012) and have intensity 100 times brighter than the previous generation of fluorescent reporter phages (Piuri et al., 2009). Unlike the LRPs (Jacobs et al., 1993), the *Φ*^2^*GFP10* reporter phage does not require exogenous luciferase substrate and can yield more stable and microscopically detectable intensive fluorescence. The presented study has evaluated the performance of this in-house fluorophage method to determine the rifampicin (RIF), isoniazid (INH), and streptomycin (STR) resistance in a high drug-resistant setting. Here we have evaluated the performance of *Φ*^2^*GFP10* phage for detecting drug resistance from *M. tuberculosis* clinical isolates, and also developed a new phage assay method using fluorescent microplate reader.

## Materials and methods

### Strains

**Figure 1:**
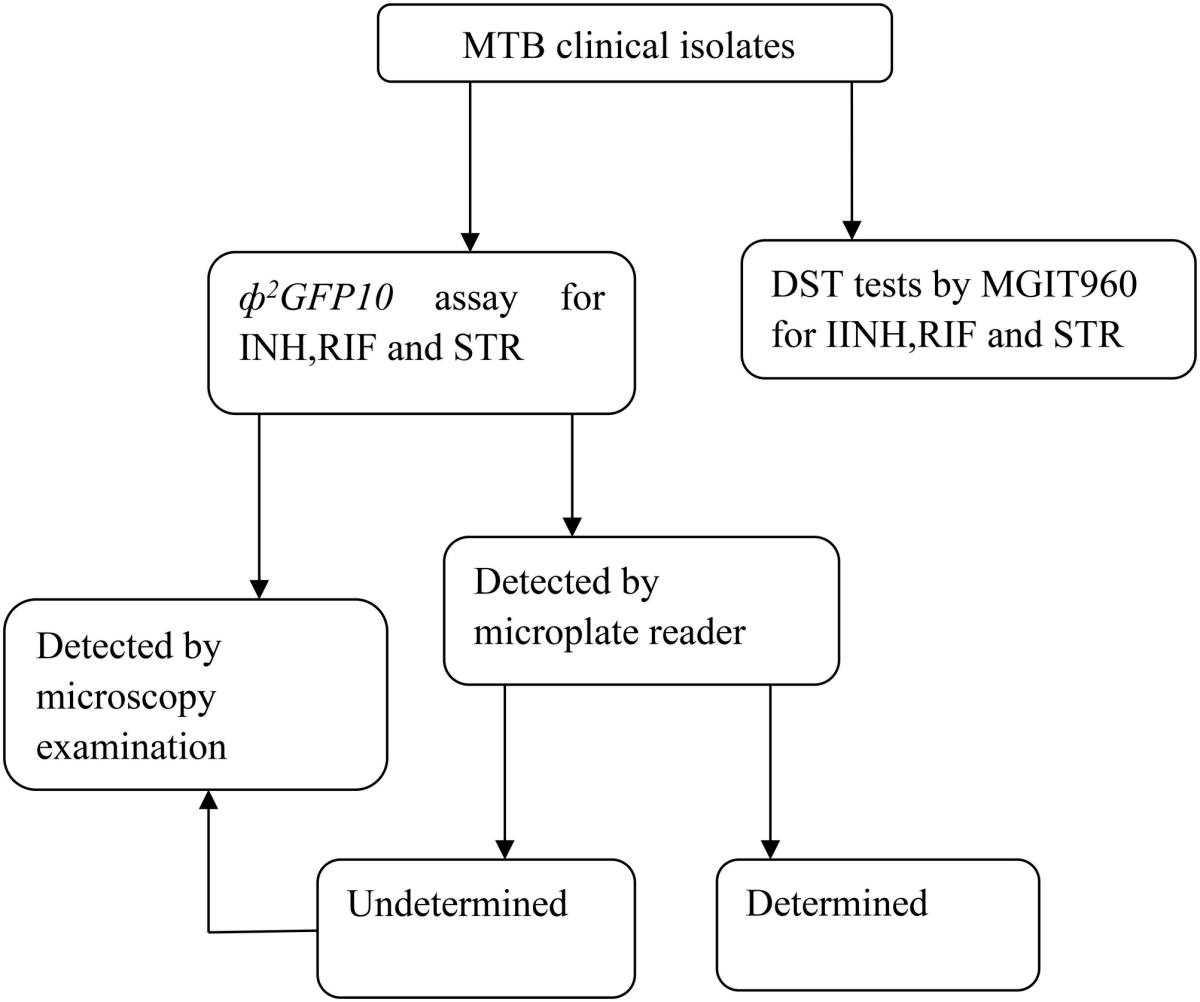
**Technique route of the study**.

A total of 128 clinical *M. tuberculosis* strains were isolated from patients with suspected drug-resistant TB patients visiting the Beijing chest hospital (Beijing, China) from April to June 2014. All of the isolates were identified as *M. tuberculosis* complex (MTBC) strains by performing a growth test on 500 μg/ml p-Nitrobenzoic Acid containing Löwenstein-Jensen medium (Tsukamura and Tsukamura, 1964). The drug susceptibility of the isolates was determined by MGIT960 SIRE kits according to the manufacturer's protocol. The critical concentrations used were as follows: INH (0.1 μg/ml), RIF(1.0 μg/ml), STR(1.0 μg/ml). Ultimately, 61, 56, and 56 were defined as resistant to INH, RIF and STR respectively by MGIT960 system. The laboratory *M. tuberculosis* H37Rv (ATCC 27294) strain was used in all batches as control.Technique round was demonstrated in Figure 1.

### Phage stock preparation

The fluorophage, *Φ*^2^*GFP10* was constructed by Dr William R. Jacobs' laboratory in Albert Einstein University (New York, USA). The stocks were prepared by growing *M. smegmatis* strain (mc^2^155) in the 7H9, containing 10% oleic albumin dextrose catalase (OADC), to an optical density of 1.0 detected by spectrophotometer at 600 nm wavelength. Then 300 μl cell suspension and 200 μl *Φ*^2^*GFP10* were mixed and incubated at room temperature for 30 min. Subsequently, 3 ml 7H9 containing 0.75% agar was added to the tube, the contents were briefly mixed and poured onto a 100-mm Petri dish containing 7H10-OADC. After the top-agar was solidified, the dish was incubated at 30°C for 48 h. Then 3 ml MP buffer [50 mM Tris (pH 7.6), 150 mM NaCl, 10 mM MgCl_2_, and 2 mM CaCl_2_] was pipetted onto the plates and then incubated on an orbital shaker for 6 h. The buffer was then collected and passed through a 0.22 μm Millipore filter membrane. The phage titers were determined by serial dilution method and the phage density was adjusted to approximately 10^10^ plaque forming unit (pfu)/ml for further use. The phage stock was stable when stored at 4°C as no appreciable drop in titer occurred for at least 6 months.

### *Φ^2^GFP10* assay by microscopy examination

INH, RIF, and STR were obtained from Sigma-Aldrich. INH and STR were dissolved in water; RIF was dissolved in DMSO. The stock solutions were 20 mg/ml, 2 mg/ml and 4 mg/ml for INH, RIF, and STR, respectively. All stock solutions were stored at −80°C during experimentation. Fresh colonies on Löwenstein-Jensen media were suspended and homogenized with sterile saline and the cell density was adjusted to 1 McFarland turbidity. A 1 ml aliquot was harvested by centrifugation and the sediment was re-suspended with 250 μl 7H9-OADC in the absence of Tween 80. The desired drugs would be added at this step, when needed, at the following finial concentrations: 20 μg/ml for INH, 2 μg/ml for RIF, or 4 μg/ml for STR. The tubes were incubated at 37°C for 24 h, or 48 h specifically for INH tests. From the stock, containing 10^10^ pfu fluorophage per ml, 100 μl volume was added to each tube to obtain a multiplicity of infection (MOI) of 100, the tubes were then incubated at 37°C for 16 h. After adding 350 μl of 4% paraformaldehyde in phosphate-buffered saline (PBS) to each tube, the reaction mixtures, left at room temperature for 90 min to ensure killing of the bacteria, were then centrifuged at 12,000 g for 15 min to remove excess phage and media. The pellet was re-suspended with 10 μl 7H9 media. Five micro liter re-suspension was spotted on a glass slide and visualized by a fluorescence microscope. The criterion for resistance with phage assay was the presence of at least one fluorescent bacillus per 50 high power fields (Rondon et al., 2011).

### *Φ^2^GFP10* assay by microplate reader

To detect fluorescence of *Φ*^2^*GFP10* in a 96 well format relative light unit (RLU) of fluorescence was measured by a fluorescent microplate reader (Berthold LB970, Germany). The wavelength of excitation and emitted light was 485 and 535 nm respectively and the counting time was 5 s. Each test was performed in triplicate and the mean value was used for interpretation. After removing excess phage and media by centrifuging, the pellet was re-suspended in 200 μl PBS, and then, the RLU of the plate was read. The influence of drug exposure on the fluorescence production of the bacilli was interpreted by the remaining fluorescence rate (RFR), calculated by the following formula: (reaction counts-blank counts)/(positive control counts –blank counts) × 100% (“reaction” indicates the well with drug exposure; “blank” indicates the well without drug and phage; “positive control” indicates the reaction without drug). Precision of the assay was determined with quintuple measurements of the following three strains: H37Rv which was susceptible to all the three tested drugs, strain 14,301 which was resistant to all the three tested drugs and strain 14,161 which was resistant to INH and STR but susceptible to RIF. The stability of fluorescence was determined by detecting the same samples at 0, 4, and 24 h, respectively. All the reactions were performed in triplicate.

### Statistical analysis

According to MGIT960 phenotypic outcomes, receiver-operation characteristic (ROC) analysis was performed using SPSS (version 17.0) and used to define a cutoff value for phage plate reader assay. Kappa value was calculated to compare the method between *Φ*^2^*GFP10* assay and MGIT960 phenotypic outcomes. Sensitivity, specificity, positive predictive value (PPV), negative predictive value (NPV) was calculated from the following URL: http://vassarstats.net. The discriminative power of fluorescent microplate reader for drug resistance was analyzed using ROC curve and area under the curve (AUC). The optimal cut-off value was defined as the one with the least (1 − sensitivity)^2^ + (1 − specificity)^2^ value (Shu et al., 2013).

## Results

### *Φ^2^GFP10* assay by microscopy

According to the outcomes of MGIT960, the sensitivity of *Φ*^2^*GFP10* assay for detecting INH, RIF and STR resistance were 100, 98.2, and 89.3% respectively whereas the specificities were 85.1, 98.6, and 95.8% respectively. The agreement between phage assay and MGIT960 for detecting INH, RIF and STR resistance was 92.2, 98.4, and 93.0%, respectively (see Table 1).The Kappa coefficient between *Φ2GFP10* assay and MGIT960 phenotypic outcomes for INH, RIF and STR were 0.85, 0.97, and 0.856, respectively.

**Table 1:** **Results and performance parameters: *Φ*^2^*****GFP10*** **assay by microscopy vs. MGIT960 system**.

**Antibiotics**	**No. of isolates**					**% (95% CI)**			
	**MGIT960 R**		**MGIT960 S**		**Total**	**Sensitivity**	**Specificity**	**PPV**	**NPV**
	**Phage R**	**Phage S**	**Phage R**	**Phage S**					
INH	61	0	10	57	128	100.0 (92.6–100.0)	85.1 (73.8–92.2)	85.9 (75.2–92.7)	100.0 (92.1–100.0)
RIF	55	1	1	71	128	98.2 (89.2–99.9)	98.6 (91.5–99.9)	98.2 (89.2–99.9)	198.6 (91.5–99.9)
STR	50	6	3	69	128	89.3 (77.4–95.6)	95.8(87.5–98.9)	94.3 (83.4–98.5)	92.0 (82.8–96.7)

R, resistant; S, sensitive.

### *Φ^2^GFP10* assay by fluorescent microplate reader

**Figure 2:**
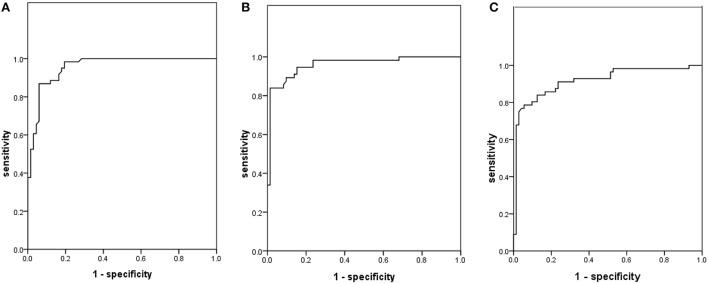
**Receiver operating characteristic curves of fluorescent microplate reader in detecting drug resistance according to BactecMGIT 960 system. (A)** INH; **(B)** RIF; **(C)** STR.

In the preliminary validation of the assay among the 3 tested isolates, the stability tests demonstrated that the fluorescence was stable for at least 24 h at 4°C (see Table 2). The coefficient of variation (CV) of RLU among the tested isolates in quintuple ranged from 1.22 to 6.73%, with the mean value at 4.67 ± 1.26%. In the validation assays using clinical isolates, in contrast to the phenotypic DST, the AUC under the ROC curve of INH, RIF and STR were 0.957, 0.960, 0.917, and the optimal cutoff value for each drug has been listed in Table 3 and Figure 2. The sensitivity of *Φ*^2^*GFP10* assay by plate reader for detecting INH, RIF and STR resistance, according to the cutoff values, were 86.9, 89.3, and 83.9%, respectively, while the specificity for INH, RIF and STR resistance detection were 94.0, 90.3, and 87.5%, respectively. The Kappa coefficient between *Φ*^2^*GFP10* assay and MGIT960 phenotypic outcomes for INH, RIF and STR were 0.73, 0.79, and 0.71, respectively. Furthermore, evaluation of the RFR value according to the susceptibility predictability demonstrated that when the RFR is lower than 45% the NPV for INH, RIF and STR were 98.0, 98.2, and 87.5%, respectively. Similarly, when the ratio was higher than 60%, the PPV for INH, RIF and STR were 92.6, 97.9, and 95.3%, respectively. When the ratio was between 45 and 60%, then the outcome should be interpreted as undetermined (see Table 4).

**Table 2:** **The stability and precision of fluorescent microplate reader assay**.

**Stains**	**Time (h)**	**The counts of** ***Fluorescent microplate reader*** **(mean**±**SD)**				
		**Blank control**	**Positive control**	INH (RFR^*^)	RIF (RFR^*^)	STR (RFR^*^)
H37Rv	0	5476.00 ± 342.39	9902.00 ± 503.95	6926.00 ± 84.44 (32.76)^*^	6296.00 ± 267.82 (18.53)	6540.00 ± 256.12 (24.04)
	4	5336.00 ± 359.35	9626.00 ± 515.10	6792.00 ± 181.30 (33.94)	6974.00 ± 323.77 (26.53)	6766.00 ± 253.24 (33.33)
	24	5218.00 ± 299.03	10088.00 ± 624.40	6414.00 ± 283.16 (24.60)	6364.00 ± 335.01 (23.53)	6476.00 ± 302.62 (25.83)
14031	0	5144.00 ± 184.07	8650.00 ± 347.20	8058.00 ± 297.19 (83.11)	8688.00 ± 413.55 (108.08)	8314.00 ± 533.32 (90.42)
	4	5140.00 ± 252.59	8410.00 ± 312.17	7992.00 ± 342.15 (87.22)	8302.00 ± 452.46 (97.70)	8352.00 ± 448.74 (98.23)
	24	5318.00 ± 332.60	8558.00 ± 257.04	8230.00 ± 278.57 (89.88)	8470.00 ± 527.4 (97.28)	8516.00 ± 310.85 (98.70)
14161	0	4568.00 ± 273.17	8354.00 ± 417.17	7694.00 ± 327.46 (82.57)	5380.00 ± 185.07 (21.45)	7612.00 ± 353.09 (80.40)
	4	4576.00 ± 298.63	8338.00 ± 363.00	7566.00 ± 458.34 (79.48)	5768.00 ± 206.57 (31.69)	7650.00 ± 401.25 (81.71)
	24	4688.00 ± 80.44	8502.00 ± 378.31	7948.00 ± 382.84 (85.47)	5320.00 ± 257.97 (16.57)	7884.00 ± 477.94 (83.80)

* Remaining fluorescence rate (RFR) = (reaction -blank)/(positive control–blank) × 100% (“reaction” indicates the well with drug exposure; “blank” indicates the well without drug and phage; “positive control” indicates the reaction without drug).

**Table 3:** **Results and performance parameters:** ***fluorescent microplate reader*** **vs. MGIT960 system**.

**Antibiotics**	**AUC**	**Cutoff (%)**	**% (95% CI)**			
			**sensitivity**	**specificity**	**PPV**	**NPV**
INH	0.957	55.00	86.9 (75.2–93.8)	94.0 (84.7–98.1)	93.0 (82.2–97.7)	88.7 (78.5–94.7)
RIF	0.960	53.60	89.3 (77.4–95.6)	90.3 (80.4–95.7)	87.7 (75.7–94.5)	91.5 (81.9–96.5)
STR	0.917	48.65	83.9 (71.2–91.9)	87.5 (77.1–93.8)	83.9 (71.2–91.9)	87.5 (77.1–93.8)

**Table 4:** **Results and performance parameters by sectional outcomes interpretation:** ***fluorescent microplate reader*** **vs. MGIT960 system**.

**Drug**	**RFR**	**No. of isolates**	**MGIT960 DST outcomes**	
			**R**	**S**
INH	<45%	51	1	50
	45–60%	23	10	13
	>60%	54	50	4
RIF	<45%	56	1	55
	45–60%	25	9	16
	>60%	47	46	1
STR	<45%	64	8	56
	45–60%	21	7	14
	>60%	43	41	2

R, resistant; S, sensitive; RFR, remaining fluorescence rate.

## Discussion

Significant efforts are being made to develop rapid, simple and accurate tests for drug- resistant *M. tuberculosis*. One of such technologies currently been worked at is based on using bacteriophages (Banaiee et al., 2008; Piuri et al., 2009; Rondon et al., 2011; Jain et al., 2012; Sivaramakrishnan et al., 2013). LRP have been used for identifying drug resistance by detecting the luminescence produced by LRP after their infection in *M. tuberculosis.* These assays classify the samples as drug-susceptible if no luminescence is detected in the luminescence drug-containing samples. A meta-analysis showed that the sensitivity and specificity of luciferase reporter phage for rapid detection of RIF resistance in *M. tuberculosis* ranged from 92 to 100% and 89 to 100% (Minion and Pai, 2010). Although this method has shown high sensitivity and high specificity, a relatively expensive luminometer is required. The newly constructed phage *Φ*^2^*GFP10* facilitates the microscopic signal detection since it yields 100-folds higher fluorescence signal per-cell than any previously described reporter phages (Jain et al., 2011). Meanwhile, as the intensive fluorescence increased the contrast between reaction and background, tedious washing steps were not necessary for phage assay, so the *Φ*^2^*GFP10* assay was easier to perform than the other phage assays (Rondon et al., 2011).

In this study, drug resistance detection by *Φ*^2^*GFP10* assay yielding applicable sensitivities and specificities, especially for RIF. A recent report also obtained great consistency between *Φ*^2^*GFP10* assay and GeneXpert for RIF resistance diagnosis (O'Donnell et al., 2015). Fluorescent microscopy may be a limiting factor for *Φ*^2^*GFP10* assay application due to its cost, whereas less expensive and simpler fluorescent microscopes using light emitting diodes (LED) have been applied for smear test of tuberculosis in various clinical laboratories (Marzouk et al., 2013; Reza et al., 2013; Xia et al., 2013) and they could readily be adapted for *Φ*^2^*GFP10* assay. The reagents for fluorophage growth and amplification are inexpensive, safe, and universally available. The total cost for the 3-drug test per strain was less than US $2. Additionally, fixation of the sample with paraformaldehyde prior to analysis overcomes the substantial biosafety concerns.

In our study, even though a longer drug exposure time of 48 h for INH, the specificity was still lower than those of RIF and STR whose drug exposure time was 24 h. Those observations were consistent with other mycobacteriophage-based assays (Rondon et al., 2011). The anti-TB mechanism of INH involves inhibiting the synthesis of mycolic acid of cell wall (Eltringham et al., 1999), which is a slow process compared with the quickly bactericidal activities of RIF and STR, therefore the bacilli can stay alive for quite a while even when they are sensitive to INH. The fluorophage could infect such bacilli and produce fluorescence signal which decreased the specificity of the assay. To enhance the bactericidal activity of INH, we increased the INH concentration for phage assay to 20 μg/ml, which was dramatically higher than those of previous studies (Xia et al., 2013; Zhang et al., 2015). However, the specificity was still lower for INH than those of RIF and STR, which indicating the INH tolerance deviation for clinical strains was universal.

First-line-drug ethambutol (EMB) also affects the cell wall construction in *M. tuberculosis*, and is a bacteriostatic drug rather than a bactericidal drug. We attempted to develop a *Φ*^2^*GFP10* assay for EMB, but the pilot test demonstrated that even an exposure concentration of 50 μg/ml could not lead to obvious CFU loss. A previous study reported low concordance (53%) between the phage amplified biologically assay and resistance ratio method for EMB, which they attributed to the bacteriostatic role of EMB both *in vitro* and in a macrophage model (Eltringham et al., 1999). Therefore, the *Φ*^2^*GFP10* assay might only be feasible to test the rapid-action bacteriocidal drugs.

Although we acquired favorable sensitivity and specificity for *Φ2GFP10* assay by microscopy, we found that microscopic examination process is experience dependent, and a lot of time is required when handing a batch of samples. So we developed a phage assay to interpret the outcomes simply by fluorescence plate reader, which can read a 96-well plate in 10 min. We monitored the fluorescence diminishing after the bacilli were exposed to the tested drugs. When categorized the RFR lower than 45% as “susceptible,” greater than 60% as “resistant,” while between 45 and 60% as undetermined, plate reader could interpret over 80% of the test strains successfully for all the 3 drugs, with 95.24% (100/105), 98.06% (101/103), and 90.65% (97/107) consistency with MGIT960 system for INH, RIF, and STR susceptibility tests, respectively. Therefore, we suggest interpreting the outcomes in sections to increase the reliability of the assay. For undetermined samples, we suggest to recheck by microscopy to produce more reliable results.

Like other fluoromycobacteriophages, *Φ*^2^*GFP10* assay could not discriminate between *M. tuberculosis* and other members of MTBC species (Riska et al., 1997). For the settings with high isolation rate of nontuberculous mycobacteria, one should be cautious to report drug resistance without MTBC identification.

In summary, the *Φ*^2^*GFP10* assay is a rapid, simple and inexpensive DST method. In clinical laboratories with limited resources, the *Φ*^2^*GFP10* based assay has the potential to be used for drug resistance diagnosis of tuberculosis.

## Author contributions

HH designed the study. HH and XY wrote the paper. WJ modified the paper. XY and YG performed the *Φ*^2^*GFP10* assay by microscopy. XY and ZS performed *Φ*^2^*GFP*10 assay by Microplate reader. GJ, YM, and LZ performed DST by MGIT960 kits. XY conducted statistical analysis. PJ, MO, and ML established the assay for phage stock preparation. All authors reviewed the results and approved the final version of the manuscript.

### Conflict of interest statement

The authors declare that the research was conducted in the absence of any commercial or financial relationships that could be construed as a potential conflict of interest.
